# Commercial Fungicide Toxic Effects on Terrestrial Non-Target Species Might Be Underestimated When Based Solely on Active Ingredient Toxicity and Standard Earthworm Tests

**DOI:** 10.3390/toxics10090488

**Published:** 2022-08-23

**Authors:** Gabriella Jorge-Escudero, Mariana Pérez Polanco, Jan Erland Lagerlöf, Carlos Alberto Pérez, Diana Míguez

**Affiliations:** 1 Departamento de Sistemas Ambientales, Facultad de Agronomía, Universidad de la República, Garzón 780, Montevideo 12900, Uruguay; 2 Department of Ecology, Swedish University of Agricultural Sciences, P.O. Box 7044, SE-75007 Uppsala, Sweden; 3 Departamento de Protección Vegetal, EEMAC, Facultad de Agronomía, Universidad de la República, Ruta 3 km 363, Paysandú 60000, Uruguay; 4 Latitud-LATU Foundation, Technological Laboratory of Uruguay (LATU), Montevideo 11500, Uruguay

**Keywords:** *Eisenia fetida*, *Glossoscolex rione*, fusarium head blight, cocktail effect, other ingredients, formulated product

## Abstract

The ecosystem services provided by earthworms are lost when land management reduces their populations, hence, the importance of thorough assessments of management effects on this group. The present study aimed to: (1) review the possible influence of other ingredients within the formulations of two commercial fungicides; (2) assess the sublethal effects of these commercial fungicides on *Eisenia fetida*; and (3) assess the acute lethal effects of one commercial fungicide on both *Glossoscolex rione* and *E. fetida*. Examining all components of the studied commercial formulations revealed that alongside the toxic active ingredients are other ingredients that are equally as or more toxic than the former and may even be in higher concentrations. The inhibition concentration of 10% of *E. fetida’s* progeny (IC_10_) was estimated at 133 mg kg^−1^ for PROSARO^®^ and 1544 mg kg^−1^ for SWING PLUS^®^. Both fungicides showed an effect of hormesis on the progeny. In this first toxicity study with *G. rione*, it was found that this species is more sensitive to PROSARO^®^ than *E. fetida*, with preliminary 14 day-lethal concentrations of 285 mg kg^−1^ for the former and >1000 mg kg^−1^ for the latter.

## 1. Introduction

Earthworms play a fundamental role in soils, contributing to their physical, chemical and biological properties. Several studies have shown that earthworms favour plant development and health as part of the ecosystem services they offer [1]. For these reasons, agricultural management practices that are detrimental to earthworm populations also affect the long-term production and productivity of the soil [2,3]. Agrochemicals can have acute effects on worms, forcing them to move away or causing their deaths, which reduces their populations in the short term. In addition, agrochemicals’ sublethal effects on growth, reproduction rate and/or offspring development may also reduce earthworm populations in the long term [4,5].

The toxicity of the active ingredients in agrochemicals (chemicals meant to kill or repel pests in crops) is tested on various animals, including earthworms, as an international requirement to elaborate safety sheets following the United Nations’ standard classification and labelling for hazardous materials within the Globally Harmonized System of Classification and Labelling of Chemicals (GHS). However, the field application of agrochemicals is carried out with commercial formulations or end-use products that may combine more than one active ingredient including ingredients that play some role other than pest control, for instance as solvents and adjuvants (chemicals added to products to help the pesticide do its job and stay on target; many adjuvants are surfactants employed to increase the penetration of the active ingredient in the leaf) [6]. Hence, these commercial formulations may have mixed toxicity effects on non-target organisms. Despite this, the other ingredients are not listed on product labels for EPA-registered pesticides because they are considered confidential business information, and they may also be listed as “inert” ingredients. Although other ingredients are sometimes called inert, the name does not mean that they are nontoxic.

Nagy et al. [7] reviewed the interactions of ingredients in pesticide formulations and found that that in several cases, the formulations have different toxicities than their active ingredients. Recent studies with enchytraeids support the idea that analysing the toxicities of end-use products, rather than active ingredients, will give “a more realistic environmental hazard of pesticides” [8]. Moreover, the complete formulations are not tested by industry applicants for long-term toxicity for regulatory purposes [9,10]. For example, the combined toxicity of insecticides, herbicides and a heavy metal was examined by Chen et al. [11] by conducting acute earthworm toxicity assays in multicomponent mixtures; the authors found that enhanced synergistic effects predominated in the majority of multi-component mixtures, concluding that this may entail implications for terrestrial risk assessment. In some cases, additives reduce toxicant availability as they may easily be bound to soil particles such as clay and organic matter [9]. In other cases, herbicides such as glyphosate exhibit greater toxicity when in mixtures with adjuvants than alone, and the active ingredient has been found not to be the major toxic compound in several herbicide formulations. Additionally, some petroleum-based compounds in herbicides proved to be significantly more toxic than glyphosate [12].

In the European Union (EU), active ingredients are authorised at the EU level under Regulation 1107/2009, while the final pesticide products are authorised at the Member State/national level. In the United States, the current regulations require submitting at least product chemistry and acute toxicity data for the product if it is not substantially similar to another registered one [13]. However, many chemicals pose different modes of action other than acute, and mixture toxicity cannot be disregarded as there might exist additive, synergistic or antagonistic modes of combined actions. New requirements introduced in 2013 to the EU regulations, based on the idea that “the toxicity of the plant protection product cannot be predicted on the basis of the data for the active substance” alone, include information regarding the toxicity of the end-use product; in the case of earthworms, the focus is on sublethal effects rather than acute effects, e.g., the no effect concentration (NOEC) or the effective concentrations for 10 and 20% (EC_10_ and EC_20_, percentages of organisms showing a specified nonlethal effect) [14]. Nevertheless, in other countries, such as Uruguay, the approval of pesticide products is still mostly limited to determining the toxicity of the individual ingredients while ignoring the possible combined effects of mixtures contained in end-use products on non-target soil organisms, not even considering earthworms [15]. According to the Guidance Document on Statistical Methods for Environmental Toxicity Tests [16], although EC_10_ and EC_20_ describe quantal effects, they are sometimes mistaken for quantitative estimators, which should actually be correctly defined as ICps, inhibiting concentrations for a (specified) percentage effect. The latter may be estimated by regression or by smoothing and interpolation).

Standardized toxicity tests for non-target soil organisms such as earthworms are performed mostly with *Eisenia fetida* or *E. andrei* given the convenience of their short reproductive cycles and their ease of breeding and management [17,18,19,20]. However, this approach has been questioned since these species, due to their ecology, are rarely found in agricultural fields where agrochemicals are actually applied; they are *epigeic* and inhabit sites with high accumulation of organic matter on surfaces such as manure [21]. In addition, *endogeic* species such as *Aporrectodea caliginosa* and *A. icterica* that do inhabit cultivated fields have been shown to be more sensitive than *Eisenia* spp. [12,13,16]. Buch et al. [22] found that the acute lethal and avoidance behaviours of *Pontsocolex corethrurus*, widespread in Brazil, are comparable with those obtained with *E. fetida*, so the latter would be a good test species in this case. The use of avoidance tests has been recommended as they are more sensitive than lethality tests [23], but in the case of native species, the former may have erratic results [24], perhaps due to lower mobility in the case of some native worms compared with *E. fetida*.

One of the native species found in northern Uruguay is *Glossoscolex rione* Ljungström, 1972. There is very little information about the ecology of this species. Cordero [25] reports its presence in Uruguay and describes morphological differences with other similar species of the same genus. There are no previous reports regarding breeding *G. rione* or using it for toxicity testing. The scarce information available reports that this endogeic earthworm had a very high survival percentage in an experiment carried out over 6 weeks in moist soil cores at 19 °C ± 2 °C [26]. Since the same author reported the presence of this species in wheat fields, it is of interest to know how it is affected by agrochemicals used in this crop to control fusarium head blight, one of the main cereal diseases in Uruguay and worldwide. These agrochemicals include mostly triazole-based fungicides which are applied by spraying at full flowering and 10 days later [27].

We hypothesized that fungicides would have a detrimental effect on *G. rione* survival and that these earthworms would be more sensitive than the standard test organisms, *E. fetida*. The aims of the present study were: (1) to study the possible influence of other ingredients within the formulations of two commercial fungicides by examining the hazards of each one at a screening level based on official information presented by the European Chemicals Agency (ECHA), the United States National Institutes of Health (NIH) and the International Union of Pure and Applied Chemistry (IUPAC) and in relevant earthworm toxicity test data found in the literature; (2) to assess the sublethal effects of these commercial fungicides on *E. fetida*; and (3) to assess the acute lethal effects of one commercial fungicide on both *G. rione* and *E. fetida*. 

## 2. Materials and Methods

### 2.1. Review of Hazard Identification

We retrieved toxicity data on each active substance, ingredient or adjuvant stated in the Material Safety Data Sheets (MSDSs) of both herbicides under test. To achieve this goal, information was obtained from the IUPAC Pesticides Properties DataBase (http://sitem.herts.ac.uk/aeru/ppdb/en/index.htm, accessed on 15 February 2022) in the case of the active ingredients and queried using the ECHA dossiers (https://echa.europa.eu/information-on-chemicals, accessed on 15 February 2022) and the open chemistry database PubChem of the NIH (https://pubchem.ncbi.nlm.nih.gov/, accessed on 15 February 2022) of the United States for the rest of the chemicals. This information was complemented with available research articles. The hazards of each chemical were examined based on our interpretation of the registered hazard category found in each of the mentioned sources. 

### 2.2. Test Facilities

Experiments were held in the laboratory of Latitud-LATU Foundation, at the facilities of the Technological Laboratory of Uruguay (LATU), Montevideo.

### 2.3. Test Organisms

Individuals of *E. fetida* were obtained from the Composting Demonstration Unit of the Faculty of Agronomy, Montevideo, and kept in a substrate of organic manure and loose peat of *Sphagnum* sp. (Kekkilä^®^, Finland) combined in the ratio 1:1 (*v*/*v*) with a water content of 70% (wet basis moisture content: *w*/*w*). Individuals of *G. rione* were collected from the Mario Cassinoni Experimental Station of the Faculty of Agronomy, Paysandú, kept in soil obtained from the same collection site with a moisture content of 20–25% (*w*/*w*) and fed with ecologically bred (organic) cow manure. All earthworms were acclimated in the laboratory cultures at 20 °C for at least one month before the tests.

### 2.4. Test Substances

Two commercial fungicides with different toxicological properties were tested: SWING PLUS, BASF^®^ (SP) (active ingredients: metconazole 27.5% and epoxiconazole 37.5% (weight/volume: *w*/*v*)), and PROSARO, BAYER^®^ (P) (active ingredients: tebuconazole 12.5% and prothioconazole 12.5% (*w*/*v*)). Cibencarb, CIBELES^®^ (active ingredient carbendazim 500 g L^−1^) was used as a positive control (reference substance); deionized water was used as a negative control. The two commercial fungicides were selected following official recommendations for fusarium head blight control. Cibencarb was chosen as a positive control since it contains carbendazim, which is the positive control suggested by the ISO standards [18].

### 2.5. Test Substrate

Artificial soil was prepared following ISO 11268 international standards with 10% air-dried peat of *Sphagnum* sp., 20% kaolin and 70% sand (*w*/*w*). Afterwards, the mix was enriched with 1% of rehydrated dried organic cow manure added as feed. CaCO_3_ was used to neutralize the mix to a pH 6.5–7. Water-holding capacity was 84% on a dry weight basis.

### 2.6. Experimental Setup

A set of 1.8 L glass containers were used as experimental units. These were filled with 600 g (dry weight) artificial soil which was wetted to 50% moisture (dry weight basis) corresponding to 60% of water-holding capacity. In order to simulate field application, each container was sprayed with 3 mL of fungicide emulsions (prepared with deionized water and the corresponding concentration of fungicide, see Table 1 and Table 2) or deionized water for control, left for 1 h under a fume hood, covered with a plastic film and left overnight for the fungicide to spread uniformly on the soil. The following day, 10 *E. fetida* or 5 *G. rione* adults and/or sub-adults (individuals with the size of an adult but a not fully developed clitellum) were added according to treatment. After the addition of the earthworms, the plastic film was perforated with a needle. Vessels were incubated at 20 ± 2 °C with fluorescent lighting of 600 lux with a 16L:8 d photoperiod (L = hours of light; d = hours of darkness).

### 2.7. Toxicity Test 1: Sublethal Effects-Growth and Reproduction

This test was performed following ISO 11268-2 international standards [19] using *E. fetida* (mean weight 368 ± 25 mg). SP and P were used in five different concentrations each, following a logarithmic series: one concentration lower by one order of magnitude than that recommended by the trade companies for field application and the concentration recommended by the trade companies; the three other concentrations were greater by one, two and three orders of magnitude (Table 1). The concentration used for Cibencarb^®^ (reference substance for positive control, based on carbendazim) was equivalent to that recommended by the company for field application. Experimental units were prepared according to Section 2.6 for 12 treatments (which included the 5 concentrations for each fungicide plus a negative and a positive control). Each treatment had 5 replicates, totalling 60 glass containers. All earthworms were adults with a developed clitellum.

The total test duration was 56 days. Weekly management consisted of adding 1% manure as feed and watering in order to maintain moisture levels. On day 28, adult worms were removed from the vessels, washed in tap water and gently dried with paper tissues. Earthworm number and fresh biomass were recorded. At the end of the experiment, offspring and cocoons were retrieved, counted and weighed.

### 2.8. Toxicity Test 2: Acute Effects-Lethality

A 14 day-lethality test was performed for both *E. fetida* (individual mean weight 336 ± 25 mg) and *G. rione* (individual mean weight 410 ± 55 mg) with P, which was, according to Toxicity test 1, the fungicide with the strongest effect on earthworms. It was tested in five different concentrations following a logarithmic series which ranged between the two highest concentrations used in the previous test (Table 2). The selected concentration of Cibencarb^®^ (2.5 mg kg^−1^ of soil), used as reference substance for positive control, was within the range suggested by ISO 11268 (1–5 mg kg^−1^ of soil) [19]. Experimental units were prepared according to Section 2.6 for 14 treatments, each with 5 replicates, totalling 72 glass containers. Treatments included the five concentrations of the fungicide, plus a negative (deionized water) and a positive control (carbendazim), for each earthworm species. All earthworms were adults (with a developed clitellum) or subadults (similar size as adults but lacking clitellum). On day 14, live worms were counted, washed in tap water, gently dried with paper tissues, and weighed.

### 2.9. Statistical Analysis

Quantitative (growth and reproduction) analyses were performed with R^®^ software. The 20% and 10% inhibition concentrations for both fungicides (IC_20_, IC_10_) were estimated by the adjustment of the dose–response curves according to the models described in Method Development and Applications [16] and presented below:

Hormesis model

$$Performance=\frac{t\left(1+h.logconc\right)}{\left(1+\left(\left(p+h.logconc\right)/\left(1-p\right)\right){\left(\frac{logconc}{ICp}\right)}^{b}\right)}$$

Gompertz model

$$Performance=exp\left\{log\left(1-p\right){\left(\frac{logconc}{ICp}\right)}^{b}\right\}$$

Logistic model

$$Performance=\frac{t}{\left(1+\frac{p}{1-p}{\left(\frac{logconc}{ICp}\right)}^{b}\right)}$$

where Performance was considered the number of juveniles. Note how the logistic model is a particular case of the hormesis model where parameter h adopts the value 0. To check the existence of an effect of hormesis, the adjustment of the different curves was compared with the Akaike information criterion (AIC), selecting the model with the lower value for this indicator. The parameters of these curves were iteratively estimated by minimum nonlinear squares, and their confidence intervals were calculated by profiling the verisimilitude.

A linear mixed model was used to assess the effects of the different concentrations on the assessed parameters (growth, number and biomass of juveniles) with concentration as a fixed factor, and block (replicate) as a random factor. When significant differences were found, LSD Fisher was used as a post hoc test. The normality of residuals was checked with Shapiro-Wilks’ test, modified by Mahibbur and Govindarajulu (1997, cited in [28]). These analyses were performed with Infostat^®^ (v.2016) software, powered by R through DCOM^®^.

The quantal (lethality) data analyses were performed with MedCalc^®^ (v.17.9.4) software. P 50% lethal concentrations (LC_50_ (14 days)) for each earthworm species, and their 95% confidence limits, were estimated with probit regression, using the method of maximum likelihood, given that in both cases, data provided at least two partial effects. Concentrations were log-transformed, and replicates were pooled.

Outliers were defined by Grubbs’ rule, as those for which the difference between the value in question and the mean, divided by the standard deviation, exceeds a critical tabulated value, −1.67, in the case of 5 replicates and a significance level of 5% [29]. Given the case, this value was replaced by the mean value of the other replicates.

## 3. Results

### 3.1. Review of Hazard Identification

The acute and sublethal toxicity levels for earthworms of the individual active ingredients in the studied commercial fungicides were classified as low or moderate according to the IUPAC database, whereas according to the classification provided by companies to ECHA in CLP notifications, they are all toxic to very toxic to aquatic life with long-lasting effects; some have also effects on human health (Table 3). Specifically, the active ingredient epoxiconazole is “suspected of causing cancer and suspected of damaging fertility or the unborn child” [30], and metconazole is “harmful if swallowed, and suspected of damaging the unborn child” [31]. The active ingredient prothioconazole is in the category “very toxic to aquatic life acute hazard and long-term hazard” [32], while tebuconazole is classified within Group C as a “possible human carcinogen” [33]. Regarding ecotoxicity, studies carried out with carp (*Cyprinus carpio*), showed that exposure to different concentrations of tebuconazol fungicide affected the health of the fish, due to the occurrence of oxidative stress [34].

In addition to the active ingredients, the commercial formulations that we used in the laboratory experiments contain other ingredients that are also categorized as hazardous to the environment (Table 4). In the case of SWINGPLUS^®^, based on the ECHA dossier registers, in terms of toxicity, the most hazardous adjuvant chemicals are the tristyrylphenol ethoxylates, a group of technical nonionic surfactants. This compound is rated a substance “toxic to aquatic life with long-lasting effects”. Further, the European registration classifies the naphtha solvents (hydrocarbons, C11–C15, aromatics) in this same category. The rest of the ingredients are assessed as less toxic by ECHA. For instance, benzyl alcohol is also not classified with respect to acute or chronic ecotoxicity as several acute aquatic studies of three trophic levels (fish, daphnid, algae) and long-term studies of two trophic levels (daphnid and algae) indicate that this compound has a low acute toxicity to aquatic organisms and low risk to the terrestrial component. However, although the surfactant oxirane, methyl, polymer with oxirane, monoisotridecyl ether, block is rated “moderately toxic”, the hazard sentences states “severely eye irritant” [35,36].

The formulation PROSARO^®^ contains one “other ingredient”, N,N-Dimethyldecanamide, which is a surfactant classified as “harmful to aquatic life with long lasting effects, causes serious eye irritation, skin irritation and may cause respiratory irritation” [37,38]. Apart from the general examination of risks, comments on specific earthworm toxicity were queried and are presented when available in Table 4. For instance, according to Moon et al. [39], in earthworms after a 14 day exposure, the LC_50_ of the soils contaminated with kerosene was 1079 mg kg^−1^. The analysis of the body residue concentrations of PAHs in the earthworms showed that the accumulation of alkyl PAHs predominated among the 12 priority PAHs. Although benzyl alcohol toxicity to soil macro-organisms could not be found in the literature, experimental K_oc_ < 5 suggest that benzyl alcohol is expected has very high mobility in soil [35].

**Table 3:** Acute lethal and sublethal reproduction toxicities for earthworms (*E. fetida*), the corresponding toxicity categories of the active ingredients in the tested fungicides and the health and environment hazard categories.

Formulation Trade Brand	Active Ingredient	CAS Number	Earthworm Acute Toxicity, 14 Day-LC_50_^a^ (mg kg^−1^)	Acute Toxicity Category ^b^ [40,41,42,43]	Sub-lethal Toxicity, 56 Day-Reproduction NOEC ^c^ (mg kg^−1^)	Earthworm Sublethal Toxicity Category ^d^ [40,41,42,43]	Hazard Category ^e^ [30,31,44,45]
SWING PLUS^®^	Metconazole	125116-23-6	>500	Moderate	>20	Moderate	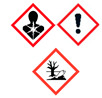
	Epoxiconazole	133855-98-8	>500	Moderate	>3.24	Moderate	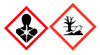
PROSARO^®^	Tebuconazole	107534-96-3	1381	Low	10	Moderate	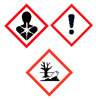
	Prothioconazole	178928-70-6	>1000	Low	1.33	Moderate	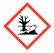

Notes: ^a^ LC_50_ = Lethal Concentration for 50% of tested earthworms. ^b^ Thresholds suggested by University of Herthfordshire for acute toxicity categorisation (mg kg^−1^): >1000 = low; 10–1000 = moderate; <10–high. ^c^ NOEC = no observable effect concentration. ^d^ Thresholds suggested by University of Herthfordshire for chronic and reproduction toxicity categorisation (mg kg^−1^): >100 = low; 0.1–100 = moderate; <0.1–high. ^e^ Pictograms according to the CLP Regulation classification and labelling system for hazardous chemicals in the European Union, which are in line with the United Nations Globally Harmonised System. 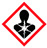: May be fatal if swallowed and enters airways. Causes damage to organs. May cause damage to organs. May damage fertility or the unborn child. Suspected of damaging fertility or the unborn child. May cause cancer. Suspected of causing cancer. May cause genetic defects. Suspected of causing genetic defects. May cause allergy or asthma symptoms or breathing difficulties if inhaled. 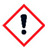: May cause respiratory irritation. May cause drowsiness or dizziness. May cause an allergic skin reaction. Causes serious eye irritation. Causes skin irritation. Harmful if swallowed. Harmful in contact with skin. Harmful if inhaled. Harms public health and the environment by destroying ozone in the upper atmosphere. 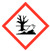: Toxic or very toxic to aquatic life with long-lasting effects.

**Table 4:** The hazards of other ingredients declared in two commercial fungicide formulations.

Formulation Trade Brand	Other Ingredient ^a^	CAS Number	Toxicity to Earthworms ^b^	Hazard Category According to ECHA ^c^	Information Source
SWING PLUS^®^	propanoic acid (2S)-2-hydroxy-2-ethylhexyl ester	186817-80-1	Not available	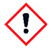	ECHA, NIH
	oxirane, methyl-, polymer with oxirane, monoisotridecyl ether, block	196823-11-7	Not available	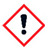	ECHA
	poly(oxy-1,2-ethanediyl), α-[2,4,6-tris(1-phenylethyl)phenyl]-ω-hydroxy-, also known as tristyrylphenol ethoxylated	99734-09-5	21-d LC_50_, earthworm *Apporectodea calignosa,* >40 mg kg^−1^. EC_50_ for growth reduction, 23.9 mg kg^−1^. EC_10_ and EC_50_ for reproduction 3.44 mg·kg^−1^ and 13.7 mg kg^−1^, respectively	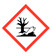	ECHA Krogh et al., 1996, cited by [46]
	naphtha solvent (oil) heavy aromatic fraction	64742-94-5	LC_50_, *E. fetida* 14 d: 129 mg kg^−1^	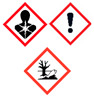	ECHA
	Kerosene unspecified	64742-81-0	LC_50_, *E. fetida*,14 d:1079 mg kg^−1^	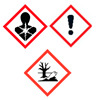	ECHA, [39]
	Benzyl alcohol	100-51-6	Not available	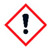	ECHA
PROSARO^®^	N,N-Dimethyldecanamide	14433-76-2	*E. fetida*, LC_50_ = 1032.1 mg kg^−1^ soil (NOEC = 562 mg kg^−1^ soil)	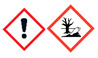	ECHA, NIH

Notes: ^a^ Other ingredient: chemical that plays some role in the commercial formulation other than controlling the pest or disease. ^b^ LC_50_ = lethal concentration for 50% of tested earthworms; EC_50_ = median effective concentration estimated to cause a specified toxic effect to 50% of the test organisms; NOEC = no observable effect concentration. ^c^ Pictograms according to the CLP Regulation classification and labelling system for hazardous chemicals in the European Union, which are in line with the United Nations Globally Harmonised System. 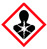: May be fatal if swallowed and enters airways. Causes damage to organs. May cause damage to organs. May damage fertility or the unborn child. Suspected of damaging fertility or the unborn child. May cause cancer. Suspected of causing cancer. May cause genetic defects. Suspected of causing genetic defects. May cause allergy or asthma symptoms or breathing difficulties if inhaled. 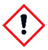: May cause respiratory irritation. May cause drowsiness or dizziness. May cause an allergic skin reaction. Causes serious eye irritation. Causes skin irritation. Harmful if swallowed. Harmful in contact with skin. Harmful if inhaled. Harms public health and the environment by destroying ozone in the upper atmosphere. 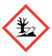: Toxic or very toxic to aquatic life with long-lasting effects.

### 3.2. Toxicity Test 1: Sublethal Effects–Growth and Reproduction

The results of this test satisfied the validity requirements of ISO 11268-2, i.e., the mortality of adults in the control was lower than 10%, and all control containers produced more than 30 juveniles. Reproduction in the negative control had one value that was identified as an outlier according to Grubbs’ rule [29]. After eliminating this value, the resulting coefficient of variance for reproduction in the negative control was 20%.

*Eisenia fetida* mortality after 28 days of exposure to the fungicides was ≤4% in the control, as well as in the 4 lower concentrations (×10^−1^ to ×10^2^) of both fungicides. The highest concentration produced 84 ± 11% and 92 ± 8% of adult mortality for SP and P, respectively. The individual biomass of surviving adults in the spiked treatments did not differ significantly (*p* = 0.618 for SP and *p* = 0.573 for P) from control (0.46 g).

**Figure 1:**
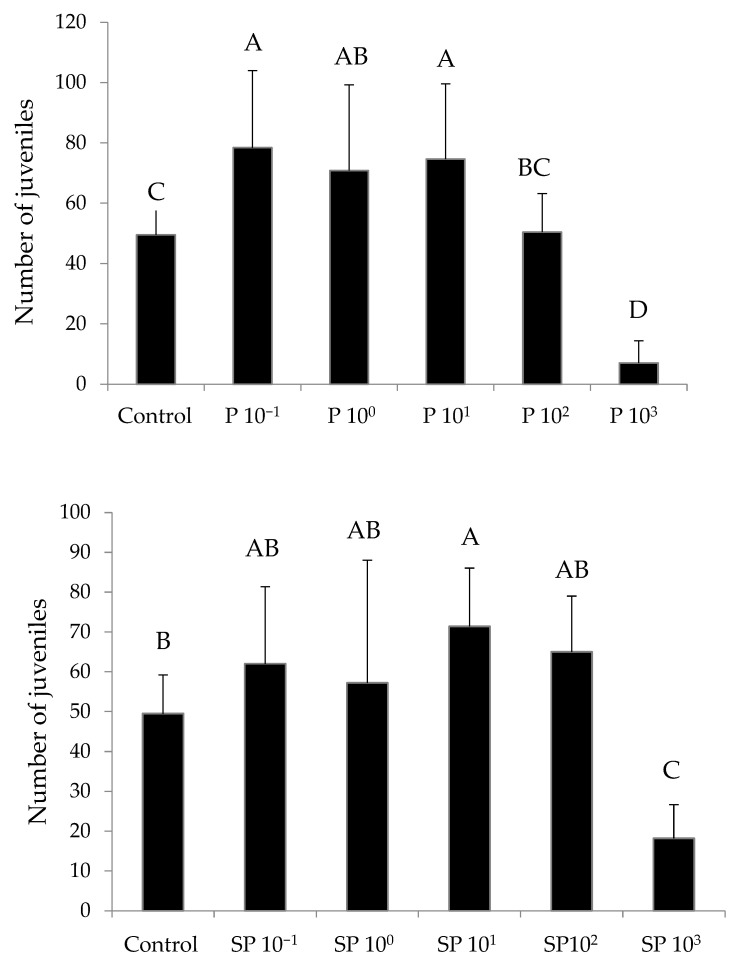
Toxicity test 1. Mean number of *E. fetida* juveniles produced during the time of the experiment per test vessel on day 56 in the presence of increasing concentrations of PROSARO^®^ (P, **above**) and SWING PLUS^®^ (SP, **below**). See Table 1 for specifications on concentrations. Error bars represent standard deviation. Means with different letters are significantly different (*p* < 0.05).

Both fungicides generated a hormetic effect, producing more juveniles at lower concentrations than in the control: up to +58% for P + 44% for SP (Table 5; Figure 1). On day 56, the estimated 20% and 10% inhibition concentrations (IC_20_ and IC_10_) for reproduction were lower for P than for SP (Table 6). Mean juvenile weight ranged from 9.79 mg to 15.37 mg in treatments with SP and from 10.00 mg to 13.91 mg in treatments with P. No treatment showed significant differences in this variable (*p* = 0.0.168 for SP and *p* = 0.918 for P) compared with the control, 12.98 ± 2.94 mg. The positive control did not show significant differences from the negative control in any of the measured variables.

### 3.3. Toxicity Test 2: Acute Effects-Lethality

**Figure 2:**
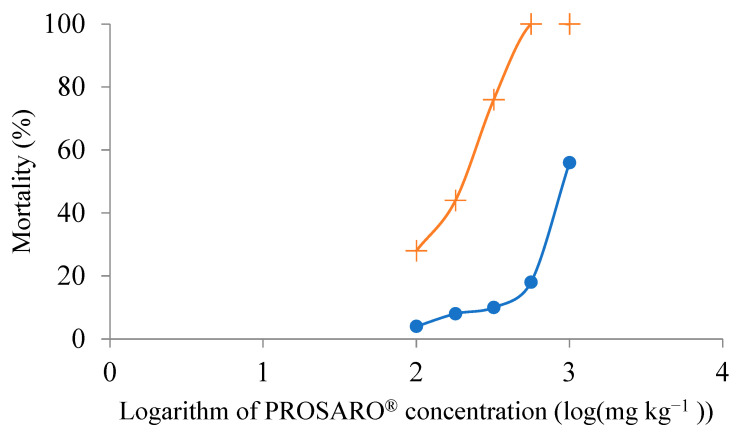
Mortality on day 14 for *E. fetida* (circle) and *G. rione* (cross) in Toxicity test 2 with increasing concentrations of PROSARO^®^, from 1 × 10^2^ to 1 × 10^3^ L ha^−1^, which were the two highest concentrations in the sublethal experiment. See Table 2 for specifications on concentrations.

The results for *E. fetida* in this test were considered valid according to ISO 11268-1 [19] since the mortality of the adults in the negative control was only 4%. The estimated LC_50_ for P with *E. fetida* at day 14 was high, >1000 mg kg^−1^ (Figure 2). The mean mortality of *G. rione* in the negative control was 52%, and there were no survivors in the two highest concentrations. *Glossoscolex rione* presented an estimated 14-day LC_50_ of 285 mg of P fungicide per kg of soil (with 223 mg kg^−1^ and 344 mg kg^−1^ as the lower and upper 95% confidence limits) (Figure 2).

Surviving *E. fetida* decreased in mean individual weight in all concentrations except for the highest, which was significantly different from the others (*p* = 0.012). On the other hand, the individual mean *G. rione* weight among the surviving individuals increased in all treatments with the addition of P, although weight did not change over time in the negative control. However, due to high variation, no significant differences in weight could be determined for this species (*p* = 0.167; Table 7). The positive control did not show significant differences form the negative control in any of the measured variables.

## 4. Discussion

Examining the hazards of all components of the studied commercial formulations revealed that alongside the toxic active ingredients occur other ingredients that are as toxic or more toxic than the former, and these may even be in higher concentrations. However, the concentrations of other components in commercial formulations is confidential information about which we can only speculate. Regarding our findings, the inhibiting concentrations of 10% and 20% in progeny (IC_10_ and IC_20_) were 2 and 3 orders of magnitude higher than the recommended field concentrations for P and SP, respectively. These sublethal effect estimators, lately recommended as superior to NOEC and LOEC (no-effect and lowest-observed-effect concentrations, respectively) [16], have mostly been used for aquatic toxicity tests [47].

The sublethal effects test covered a very broad concentration range (10,000-fold) and was used as a first screening. Hence, 28 d mortality results lacked intermediate values, jumping in the last 2 concentrations from almost 0% to close to 100%. However, in terms of reproduction, the chosen concentrations allowed for the observation of the hormetic effect on reproduction caused by both fungicides after 56 days’ exposure. Hormesis, i.e., the fact that a variable which is inhibited by high concentrations of fungicide is also stimulated by low concentrations, resulting in a biphasic (or U-shaped) concentration-response relationship, should not be regarded as a flaw but merely as a natural phenomenon defined already in the 19th century and even detected 3 centuries before [48,49]. The hormesis observed for both fungicides fell within the range of maximum stimulation averages (+30% to +60%, compared with control) reported by Calabrese and Baldwin [48].

Domínguez et al. [4] found that mean juvenile weight was significantly lower in their highest dose of AMPA (aminomethylphosphonic acid, one of glyphosate’s main metabolites found to be persistent in soils) compared with the control. They argued that this could represent a long-term negative effect as descendant earthworms would be weaker and less able to provide their ecosystem services. In the present tests, no significant differences in weight were found after 14 or 28 days for exposed adults or for surviving juveniles after 56 days of incubation. Although high variation played a role in hindering the differentiation between groups, biomass is a less-independent variable in the presence of certain mortality rates: Either all the earthworms die and no final biomass data can be registered, or a subsample of the earthworms die, and it is very likely that the weaker would die first, skewing the biomass average in favour of the more robust ones. Factors such as amount of food, or toxicant, per earthworm can also influence the mean individual biomass, either favouring or disfavouring a reduced number of earthworms [16]. In the present experiment, food availability could be discarded as a limiting factor since food surpluses were observed in most of the vessels.

The mortality results from the first screening provided criteria for selecting the logarithmic series of concentrations for the acute lethality test, as it was found that they should range between the two highest concentrations of the previous test (100–1000 L ha^−1^). Although almost 100% mortality was reached for *E. fetida* in the highest concentration of the first test after 28 days, the second test, intended to be acute, measured mortality after a shorter period (14 days), and mortality for the same concentration was lower, slightly above 50%; LC_50_ was reported as >1000 mg kg^−1^ (>1000 L ha^−1^).

Evidently, commercial fungicide acute toxicological effects are underestimated if assessed based the toxicity of their active ingredients. If these effects had been additive, neither of the commercial fungicides would have reached a concentration in this test containing LC_50_, according to the active ingredients’ concentrations in the commercial fungicide and their reported individual acute lethal toxicities (Table 3). However, almost 100% 28 day lethality was observed in the first test for both fungicides at the highest concentration. In the second test, 14 day LC_50_ for P was reached at the highest concentration in the case of *E. fetida,* which is the model organism for the standard toxicity test. Specifically, the highest concentration of P contained the amount of each active ingredient equivalent to one fifth of the reported LC_50_ (see Table 2 and Table 3). Had the effects been additive, and/or solely caused by the active ingredients, we would have expected this highest concentration to be two fifths of the LC_50_. Yet, more than 50% of the earthworms died. In previous studies with enchytraeids, P was shown to be more toxic than its active ingredients [8]. Therefore, either the effects of the two active ingredients in P have a synergistic deleterious effect on earthworms or the other components of this commercial fungicide are also affecting earthworm survival. Mesnage and Antoniou [50] highlight the influence of adjuvants such as surfactants in the increase of toxicity of active ingredients of pesticides. Nevertheless, cause-effect relationships are not easy to derive. For instance, while the chronic toxicity estimator NOEC of N,N-Dimethyldecanamide is <1000 mg kg^−1^ (562 mg kg^−1^ soil) according to ECHA [38], due to its short half-life (<6.5 h) in soil, long-term exposure to soil macroorganisms is not expected to occur.

Based on active ingredient sublethal toxicity for reproduction (Table 3), we expected to observe negative effects from the third concentrations of each commercial fungicide (SP10^1^ and P10^2^), but in the results of this experiment, only the highest concentration presented an observable negative effect. Hence, IC_20_ was higher than expected. Mismatches can also be attributed to the fact that two different estimators are being compared (NOEC and IC_20_). Recent studies have reported that tebuconazole, one of the active ingredients in P, even in low concentrations (5 mg kg^−1^) affects mRNA expression and metabolism related to earthworms’ reproductive process [51]. Although metabolomics enables researchers to detect these effects after only 7 days of exposure, measurable effects on reproduction may be evident in the long term.

To the best of our knowledge, this is the first report of a toxicity test performed on South American native earthworms of the Glossoscolecidae family. Due to the high mortality of *G. rione* obtained in the control treatment, the lethality results of P for this species must be considered preliminary, and further studies should be carried out for corroboration. The high mortality in the control could be attributed to the fact that these individuals were collected from the field, and from different sites, and not bred in the laboratory. Hence, future efforts should be directed at adjusting laboratory breeding and survival conditions for this species and assessing levels of acceptance of the artificial soil. The previous study which reported a high survival of *G. rione* in experimental conditions for several weeks had used natural soil in the experiment [26].

Species-specific sensitivity in earthworms has been suggested by Suthar [52], as their results indicate that a surface-dwelling mode of life may guard epigeic tropical earthworms against exposure to the pesticide methyl parathion, while endogeic species appeared more sensitive to the pesticide. In line with this, the present preliminary results suggest that this native temperate endogeic earthworm may also be more sensitive to the tested fungicide than the standard test earthworm, with its LC_50_ being one order of magnitude lower than that found for *E. fetida*. Although it is not confirmed yet, this is an issue to highlight, especially concerning native fauna conservation. Aware of the fact that different species may differ in their tolerance to toxicants, Kuperman et al. [53] suggest that in order to gain ecological relevance, ecotoxicological research should “include species that are geographically and ecologically representative of the location and conditions at the site”. Testing alternative earthworms found that *Ponthoscolex corethrurus* (*Pontoscolecidae*) was equally as sensitive as *E. andrei*, while *Perionyx excavatus* (*Megascolecidae* family) was less sensitive, in avoidance and/or lethality tests [22,24], whereas *L. terrestris* and *A. caliginosa* and *A. icterica* were more sensitive than the standard species [21,54].

No effect was observed in the positive control on the sublethal test, in which a field concentration was used, or on the lethal test, where a concentration within the range suggested by ISO standards was used. Although this second concentration was 6 times greater than that used in the previous test, it was still below the IUPAC 14 day LC_50_ for carbendazim (5.4 mg kg^−1^). It is important to note, though, that in these experiments, carbendazim was applied as Cibencarb (Ci), a commercial mix with 500 mg L^−1^ of carbendazim. There might have been a cocktail effect which could either emphasize or mitigate the effect of the isolated toxic substance [55].

In our experiments, where field concentrations of SP, P and Ci had no negative effect on earthworm survival, growth or reproduction, assessment was restricted to a limited time (14, 28, or 56 days) and to one single application of one single fungicide. However, in the field during a crop cycle, more than one application of the same fungicide occurs, and fungicides have proved to increase in toxicity to earthworms when the frequency of application increases [56]. In addition, the application of fungicides will likely be combined with a whole set of other pesticides (insecticide and herbicides). Interactions of these agrochemical applications have proved to decrease earthworm activity [57], and they are also expected to increase the long-term residual contamination of agricultural soils. The cocktail effect on earthworms produced by all the pesticides applied for crop production needs to be addressed in long-term studies to reach a more accurate estimation of the effect on earthworms of chemical management as a whole [5].

## 5. Conclusions

From the results of our tests with *E. fetida,* we conclude that the toxicity to earthworms of commercial fungicides may be far higher than the sum of the toxicities of their active ingredients assessed individually. Regarding the toxicity effect of the tested fungicide on *G. rione*, although there is still high uncertainty, the preliminary results suggest that this native species might have a higher sensitivity to this agrochemical compared with the standard test organism. Further research with *G. rione* should aim to obtain consistent results and confirm the bioassay application. These results are promising and are important in raising awareness of how the toxicity of agrochemicals is currently underestimated, despite all the regulation and standardizing efforts. The present study has provided new insights into the assessment of commercial products, which is important to study beyond solely the toxicity of active ingredients.

## Figures and Tables

**Table 1:** Test substance and active ingredient concentrations for treatments with SWING PLUS^®^ and PROSARO^®^ in Toxicity test 1.

Treatment ^a^		SP 10^−1^	SP 10^0^	SP 10^1^	SP 10^2^	SP 10^3^	P 10^−1^	P 10^0^	P 10^1^	P 10^2^	P 10^3^
Commercial fungicide concentration in soil	(mg kg^−1^) ^b^	0.25	2.50	25.00	250.00	2500.00	0.17	1.67	16.70	167.00	1670.00
	(L ha^−1^) ^c^	0.15	1.50	15.00	150.00	1500.00	0.10	1.00	10.00	100.00	1000.00
Metconazole	(mg kg^−1^) ^d^	0.01	0.07	0.69	6.88	68.75					
Epoxiconazole		0.01	0.09	0.94	9.38	93.75					
Tebuconazole							0.02	0.21	2.08	20.83	208.33
Protioconazole							0.02	0.21	2.08	20.83	208.33

Notes: ^a^ SP10^0^ and P10^0^ indicate recommended application doses. ^b^ Mass of commercial fungicide per mass of soil. ^c^ Volume of commercial fungicide per hectare, calculated considering 5 cm depth and a mean bulk density of 1.2 mg m^−3^. ^d^ Mass of active ingredients per mass of soil. SP = SWING PLUS^®^; P = PROSARO^®^.

**Table 2:** Test substance concentration for treatments with PROSARO^®^ (P) and the earthworm species *E. fetida* (E) or *G. rione* (G) in Toxicity test 2.

Treatments with *E. fetida*		E P 1	E P 1.8	E P 3.2	E P 5.6	E P 10
Treatments with *G. rione*		G P 1	G P 1.8	G P 3.2	G P 5.6	G P 10
Commercial fungicide concentration in soil	(mg kg^−1^) ^a^	164	295	524	916	1637
	(L ha^−1^) ^b^	100	180	320	560	1000
Tebuconazole	(mg kg^−1^) ^c^	20.83	37.50	66.67	116.67	208.33
Protioconazole		20.83	37.50	66.67	116.67	208.33

Notes: ^a^ Mass of commercial fungicide per mass of soil. ^b^ Volume of commercial fungicide per hectare, calculated considering 5 cm depth and a mean bulk density of 1.2 Mg m^−3^. ^c^ Mass of active ingredients per mass of soil. P = PROSARO^®^; E = E. fetida; G = G. rione.

**Table 5:** Akaike information criteria (AIC) to determine the best-fitting model to estimate the inhibition concentration quantitative end-points based on results from Toxicity test 1.

AIC	PROSARO^®^	SWING-PLUS^®^
Hormetic model	275.82	272.91
Gompertz model	281.25	275.15
Logistic model	283.96	276.30

**Table 6:** The 20% and 10% inhibition concentrations (IC_10_ and IC_20_) and estimations (IC95%) for two fungicide formulations considering performance in terms of number of juveniles. Based on results from Toxicity test 1.

	PROSARO^®^ mg fungicide kg Soil^−1^	SWING-PLUS^®^ mg fungicide kg Soil^−1^
IC_10_	133.3 (20.13–1776)	1544 (153.9–>>9000)
IC_20_	233.3 (35.95–3269)	2987 (327.8–>>9000)

**Table 7:** Toxicity test 2. Mean individual biomass of *Eisenia fetida* and *Glossoscolex rione* (increase or decrease relative to biomass at start) (±SD) after 14 days’ exposure to PROSARO^®^ at different concentrations and in negative control (0 L ha^−1^).

Commercial Fungicide Concentration	Mean Biomass Variation in Surviving Earthworms ^a^			
(L ha^−1^)	(%+SD)			
*E.* *fetida*				
0	−0.37 *	±	1.48	A
100	−0.90	±	1.55	A
180	−1.32	±	2.77	A
320	−0.59	±	1.08	A
560	−0.33	±	3.19	AB
1000	0.98	±	2.83	B
*G.rione* ^b^				
0	−1.23	±	3.57	
100	5.35	±	2.98	
180	6.36	±	11.24	
320	4.78	±	15.47	

Notes: ^a^ Different letters denote significant differences among treatments. ^b^ No survivors of *G. rione* in concentrations > 320 L ha^−1^. * *p* < 0.05.

## Data Availability

Not applicable.
